# Transcriptomic Profiling of Canine Testicular Leydig Cell Tumors Uncovers Key Upregulated Gene Pathways

**DOI:** 10.3390/ani16132005

**Published:** 2026-07-01

**Authors:** Malgorzata Kotula-Balak, Recep Uyar, Emilia Morańska, Grzegorz Lonc, Ummu Gulsum Boztepe, Wojciech Lopuszynski

**Affiliations:** 1 Department of Basic Sciences, Faculty of Veterinary Medicine, University of Agriculture in Krakow, Al. Mickiewicza 24/28, 30-059 Krakow, Poland; grzegorz.lonc@urk.edu.pl (G.L.); gulsumbztp@gmail.com (U.G.B.); 2 Genetic Department, Institute of Forensic Science, Ankara University, Ankara 06520, Türkiye; uyarr@ankara.edu.tr; 3 Integrated Technologies Research Center, Ankara 06790, Türkiye; 4 Department of Plant Biology and Biotechnology, Faculty of Biotechnology and Horticulture, University of Agriculture in Krakow, Al. 29 Listopada 54, 31-425 Krakow, Poland; emilia.moranska@urk.edu.pl; 5 Department of Pathomorphology and Forensic Veterinary Medicine, University of Life Sciences in Lublin, 20-950 Lublin, Poland; wojciech.lopuszynski@up.edu.pl

**Keywords:** dog, next generation sequencing, Leydig cell tumor, PI3K-Akt pathway, angiogenesis, lipid homeostasis, estrogens

## Abstract

Total RNA was isolated from sections of healthy testes and Leydig cell tumors of mixed-breed dogs using TMA Master II device. The RNA-seq libraries were sequenced on the Illumina platform. Following differential expression analysis, Gene Ontology (GO), Kyoto Encyclopedia of Genes and Genomes (KEGG), and Gene Set Enrichment Analysis (GSEA) were applied with quality control obtained using FastQC and Trimmomatic. This analysis revealed 1500 transcripts, including 928 upregulated and 168 downregulated genes. The results demonstrated that a significant proportion of these differentially expressed genes are directly involved in the control of sex steroid production (*CYP11A1*, *STAR*, and *3β-HSD3B1*) or tube formation, angiogenesis, and extracellular matrix remodeling in interstitial cells (*ESM1*, *FGG*, and *VEGFA*). Moreover, we identified the upregulation of transcripts responsible for neurotransmitter or neuroendocrine signaling (*SLC6A4*, *GRIN2C*, *GABRB3*) and cholesterol metabolism and its regulation (*GPX3*, *MSMO1*, *DHCR24*). These genes were strongly associated with the phosphatidylinositol-3-kinase (PI3K)-Protein Kinase B (Akt) cascade and extracellular matrix interactions, features shared with various malignancies. Alterations in estrogen and relaxin signaling appear to be distinctive, understudied mechanisms specific to canine Leydig cell tumors. Concurrently, downregulated genes (e.g., *DMRTC2*, *SEMA3C*, *ALOX12*) were linked with cell differentiation, signaling and immunoregulatory pathway suppression involved in tumorigenesis. A complex transcriptomic profile of canine Leydig cell tumors was developed, revealing a conserved oncogenic core shared in some aspects with human malignancies alongside unique species-specific alterations. Findings seem to be useful for identifying novel diagnostic biomarkers and targeted therapies in veterinary oncology, establishing canine reproductive tissues as a valuable comparative biomedical model for research in human.

## Simple Summary

In this study, for the first time, transcriptomic analysis of canine Leydig cell tumors revealed 1500 differentially expressed genes, including 928 upregulated and 168 downregulated transcripts. Transcriptomic profiling was performed using RNA sequencing of formalin-fixed paraffin-embedded testicular tissues of healthy and Leydig cell tumor canine testes. Following differential expression analysis, Gene Ontology (GO), Kyoto Encyclopedia of Genes and Genomes (KEGG), and Gene Set Enrichment Analysis (GSEA) were applied to identify dysregulated biological processes and signaling pathways. The analysis highlights key molecular pathways that drive tumor growth, specifically those controlling sex steroid production, blood vessel formation (angiogenesis), tumor microenvironment remodeling, and cholesterol metabolism. Crucially, the findings show significant similarities to human Leydig cell tumor mechanisms (the activation of the PI3K-Akt cell signaling pathway) while simultaneously identifying unique, previously understudied alterations in estrogen and relaxin signaling specific to dogs. Furthermore, the downregulation of genes responsible for cell differentiation and immune defense underscores how these tumors avoid normal cellular controls. In conclusion, these discoveries provide, for the first time, foundational molecular data that can improve canine oncological early diagnostics (e.g., biomarkers and targeted therapies), while establishing canine tissues as a valuable comparative model for studying testicular cancers in humans.

## 1. Introduction

The two functions of the testis are located in two anatomical compartments. These are differentiation and release of mature spermatozoa, and the secretion of sex steroid hormones. During embryonic development, the formation of seminiferous tubules with somatic Sertoli cells determines the presence, species-specific number, and distribution of the Leydig cells of the interstitial tissue, which surround the tubules [1]. In most mammalian males, excluding humans and pigs, two populations of Leydig cells (fetal and adult) develop and are the principal sources of androgens [2,3]. The fetal population provides androgens for fetal masculinization. Specific to humans and boars, an additional perinatal population appears not to be involved in the production of androgens at high physiological levels [4]. The adult population of Leydig cells begins to develop before puberty and reaches full functionality just before adolescence. Its essential role is the completion of the masculinization of the male phenotype and the maintenance of adult male characteristics and physiology. In mammalian males, puberty is initiated by activation of the hypothalamic–pituitary–gonadal (HPG) [5]. Pulsatile gonadotropin-releasing hormone (GnRH) secretion from the hypothalamus stimulates gonadotropes in the anterior pituitary to produce the phasic gonadotropins. Luteinizing hormone (LH) binds to receptors on the membrane of Leydig cells, regulating steroidogenesis, while follicle-stimulating hormone (FSH) receptors are expressed by the Sertoli cell membrane to control spermatogenesis. In the adult mammalian male, both processes are instrumentally controlled via negative feedback to the hypothalamus and pituitary gland, with the main involved hormones: testosterone and the product of Sertoli cells, inhibin. Studies by Sprujti et al. [6] revealed that in dogs subjected to chemical castration with GnRH, the increase in LH, but not in FSH, takes place, indicating a differential regulation of the release of these gonadotrophins. It should be noted that estrogens, being metabolites of androgens, are also involved in the critical local regulation of spermatogenesis [7]. Consistent with findings in humans, boars, equines, and experimental rodents, and according to our research, estrogen and estrogenic compounds can activate different types of estrogen receptors in the canine testis [8]. Males become infertile in the absence of estrogen action, according to both clinical data and animal experiments [9,10,11]. The Sertoli cell, which also promotes androgen synthesis, is the primary source of estrogens before male puberty [12]. All of the active steroidogenic enzymes needed for the production and secretion of sex hormones are not expressed by Leydig cells during this time [13,14]. Furthermore, our current findings in dogs reveal that testicular cells locally synthesize adipokines (adipose tissue hormones), which play a supportive role in modulating both steroidogenesis and spermatogenesis [15]. Multistep Leydig cell morphological and biochemical differentiation is related to the levels and quality of secreted androgens and the control of chorionic gonadotropin in the fetus and by LH after birth [16]. Fully developed Leydig cells of the individual populations are mitotically inactive, unlike somatic cells of seminiferous tubules, which stop proliferation after the blood-testis barrier is constituted [17]. When the number of Leydig cells is constant, full functionality via production of another circulating hormone, insulin-like protein 3 (INSL3), is present [18]. This hormone controls the first phase of testicular descent in the fetus by directing the development and shortening of the gubernaculum [19]. In the adult male, INSL3 is also involved in promoting spermatogenesis [20]. Constant level of circulating INSL3 is chronically modulated by LH, or its lack [21]. In ageing males, INSL3 levels decline gradually, reflecting a decline in Leydig cell functional capacity and/or numbers [22]. It is unclear whether fetal Leydig cells degenerate, differentiate into the adult Leydig cell population, or remain in the mature gonad, thus being a target of the environmental endocrine-disrupting chemicals [23,24].

In the past, dogs and, nowadays, canine tissues, due to similarities in size, function, the prevalence of spontaneous diseases (e.g., epilepsy, adrenal, kidney, heart, or prostate diseases), and clinical symptoms, have been utilized in human studies as models [25,26,27,28]. Of note, canine reproductive genetics, physiology, and hormonal regulation share many similarities with humans. In veterinary practices, diagnostic techniques used for humans are applied [29,30,31,32,33,34]. However, many inter-breed limitations in anatomy, metabolism, and genetics still need to be considered with caution. In recent years, the vast body of evidence shows that environmental chemicals adversely affect human health and wildlife, livestock, and accompanying animal health [35,36,37,38]. In response to, e.g., environmental pollution, the One Health approach initiatives were developed to protect and improve the interconnected vulnerabilities of humans, animals, and the environment. Dogs living with humans constitute a sentinel species for environmental effects on human fertility due to exposure to the same environmental and lifestyle factors throughout life [39,40,41]. Especially, reproductive development and reproductive function perturbations, including well-recognized in men testicular dysgenesis syndrome (including hypospadias, cryptorchidism, low quality and quantity of spermatozoa, or cancers) related to decreased function of Leydig cells are documented [42,43,44,45,46,47]. Nowadays, dogs and cats are more often than before diagnosed with tumors and cancers, including those of the testis [48,49,50]. Germ cell tumors (seminomas) and somatic cell tumors: Sertoli cell tumors and Leydig cell tumors account for about 90% of cases affecting the testis [51,52]. Only a more in-depth exploration of the interplay between genetic, environmental, and demographic factors will enhance understanding, inform strategies for prevention, early detection, and treatment of testicular tumors in dogs [42]. Canine Leydig cell tumors are generally benign, while malignant Leydig cell tumors are reported to be very rare [53]. It should be noted that canine Leydig cell tumors can be associated with cryptorchidism [54]. Histopathologically, these tumors are recognized by marker protein expression (vimentin, cytokeratin, KIT protein (CD117), α-fetoprotein, β-catenin, inhibin A, melanoma, anti-Müllerian hormone, LH, and neuron-specific enolase) [55,56]. Recently, preputial cytological approaches in dogs with Leydig cell tumors have identified marked keratinization with high proportions of superficial cells, moderate to low numbers of intermediate and parabasal cells, and neutrophils that gradually decrease after removing the tumor (estrogen source) [55]. The genetic data may be helpful for further understanding tumor biology on cellular and molecular levels. In both humans and dogs, Leydig cells require improved early diagnostic and successful treatment approaches, as the first detected and commonly existing spermatogenesis disturbances are regularly diagnosed and reported in our and other studies [8,57,58].

The investigation was undertaken due to the lack of knowledge on canine Leydig cell tumor etiopathology, while mixed-breed dogs exhibit the highest diversity for such analysis. Taking into account species-specific features, as well as current anthropogenic threats to reproductive function in animals and humans that lead to common, increasing reproduction problems, it is justified. Using next-generation sequencing, we compared healthy canine Leydig cells and tumor Leydig cells to identify genes, signaling pathways, and their interactions that contribute to the etiology of the disease. Partial emphasis is placed on comparisons with molecularly well-studied human Leydig cell tumors [59] and on the identification of shared mechanisms of tumorigenesis/cancerogenesis.

## 2. Materials and Methods

### 2.1. Samples

For the study, hematoxylin-eosin (HE) stained remaining archival testicular tissue slides from corresponding paraffin blocks (the collection of the Department of Pathomorphology and Forensic Veterinary Medicine, Faculty of Veterinary Medicine, University of Life Sciences in Lublin, Poland). They were saved in the frame of the practices of academics-veterinarians in the Faculty Veterinary Clinic during the surgical castration of 3–5-year-old mature mixed-breed dogs (six dogs with healthy testes and six dogs with diagnosed Leydig cell tumors). The inclusion and exclusion criteria involved: only tumors located within the scrotal testis (those within the retained testis or with malignancy signs were excluded) and no other clinical history. The serial tissue slides were carefully analyzed to select several areas from healthy (normal) or tumor Leydig cell populations using tissue microarray (TMA) extraction. To prevent RNA degradation, all procedures were performed under RNase-free conditions. Briefly, selected areas representative of normal Leydig cells (*n* = 6) and those with Leydig cell tumor (*n* = 6) were scanned to digital form using a Panoramic MIDI scanner (3-DHISTECH, Budapest, Hungary). Areas presenting hemorrhage, inflammation, or necrosis were strictly excluded from further investigations. The marked areas were then transferred using computer software onto donor blocks and automatically extracted as 0.6 mm samples using the TMA Master II device (3-DHISTECH, Budapest, Hungary) into separate PCR tubes.

### 2.2. RNA Extraction and Amplification

Total RNA was isolated using the Quick-RNA FFPE Miniprep Kit (Zymo Research Corp., Irvine, CA, USA). RNA extraction and library preparation were performed according to the manufacturers’ protocols (Zymo Research, Irvine, CA, USA). RNA concentration and integrity were evaluated using a NanoDrop spectrophotometer (Thermo Fisher Scientific, Waltham, MA, USA) and an Agilent 4200 TapeStation (Agilent Technologies, Carlsbad, CA, USA) before library construction. The library preparation workflow included ribosomal RNA depletion, RNA fragmentation, cDNA synthesis, adapter ligation, and PCR amplification according to the manufacturer’s instructions. Samples were deparaffinized at 55 °C for 1 min. Then, protease digestion was performed at 55 °C for 2 h. To reverse crosslinking, samples were incubated at 65 °C for 15 min. Total nucleic acid purification was performed according to the manufacturer’s protocol. Briefly, the lysed tissue sample was mixed with an equal volume of 100% ethanol and transferred to a Zymo-Spin™ IICR Column (Zymo Research Corp., Irvine, CA, USA). DNase I treatment was carried out on-column, followed by washing with DNA/RNA Prep Buffer and two washes with DNA/RNA Wash Buffer (Zymo Research Corp., Irvine, CA, USA). RNA was eluted in 30 μL of DNase/RNase-Free Water (Zymo Research Corp., Irvine, CA, USA). RNA concentration was measured using a NanoDrop spectrophotometer (Thermo Fisher Scientific Inc., Waltham, MA, USA). RNA integrity was assessed with the Agilent 4200 TapeStation. Purified total RNA was used immediately for cDNA synthesis or stored at −80 °C until further use.

### 2.3. Library Preparation and NGS

RNA-Seq library was prepared using Zymo-Seq RiboFree Total RNA Library Kit with 200–300 bp insert size (Zymo Research Corp., Irvine, CA, USA) and following the protocol provided with the kit. Only RNA samples meeting the manufacturer’s recommended input and quality requirements were included for library preparation and sequencing. Specifically, samples yielding at least 250 ng of total RNA and demonstrating sufficient RNA quality, as assessed by the Agilent 4200 TapeStation analysis, were selected, whereas samples with inadequate RNA quantity or poor RNA integrity were excluded from downstream processing. For library preparation, 250 ng of total RNA from each sample was subjected to random priming, denatured, and renatured to form rRNA-cDNA hybrids, then enzymatically depleted and ligated to both adapters. Ligated DNA was amplified with Zymo-seq UDI primers and Zymo amplification premix for 11 cycles (Zymo Research Corp., Irvine, CA, USA). Post-amplification cleanup was performed with 0.8X Select-a-Size MagBeads. Finally, library quality and quantity were analyzed by Agilent TapeStation 4200 and Qubit 3.0 Fluorometer (Thermo Fisher Scientific Inc., Waltham, MA, USA). 150 bp PE (paired-end) reads were sequenced on an Illumina Novaseq X Plus sequencer (Illumina Inc., San Diego, CA, USA). The extraction and sequencing were procured and managed via the NGS service Genohub.com (https://genhub.com).

### 2.4. Data Analysis

Quality control of raw sequencing reads was performed using FastQC (v0.11.9), followed by adapter and low-quality base trimming with Trimmomatic (v0.40). Clean reads were aligned to the Canis lupus familiaris reference genome (CanFam3.1) using STAR, and gene-level quantification was performed with RSEM (v1.2.15). Mapping statistics were evaluated to assess sequencing quality, with alignment rates exceeding 87% for all samples. Differential gene expression analysis was carried out in R (v4.3) using the DESeq2 package (v1.40.2). 

Principal component analysis (PCA), volcano plots, and heatmaps were generated using the ggplot2 (4.0.3), ggfortify (0.4.19), and pheatmap (1.0.13) packages. Functional enrichment analyses, including Gene Ontology (GO), Kyoto Encyclopedia of Genes and Genomes (KEGG), and Gene Set Enrichment Analysis (GSEA), were performed using clusterProfiler (v4.8.1), msigdbr (v26.1.0), and enrichplot (https://github.com/YuLab-SMU/enrichplot, accessed on 21 June 2026). Representative enrichment plots, dot plots, cnetplots, and pathway-specific heatmaps were generated for biologically relevant pathways, including steroidogenesis, PI3K–Akt signaling, angiogenesis, and extracellular matrix remodeling. All analyses were performed using reproducible R scripts. Raw sequencing data have been deposited in the NCBI BioProject database under accession number PRJNA1428179.

## 3. Results

### 3.1. RNA-Seq Analysis

High-throughput RNA sequencing generated robust and consistent datasets across all twelve samples (for clarity of data presentation, 6 samples were used as representative: *n* = 3 control and *n* = 3 tumor). Each library yielded between ~14 and 36 million reads, with high alignment efficiency to the CanFam reference genome (87–94% mapped reads). The majority of mapped reads localized to intronic and intergenic regions, consistent with total RNA sequencing from testicular tissue, while coding and UTR-derived reads constituted a smaller but stable fraction across samples. Ribosomal RNA contamination was minimal (<0.6% in all libraries). Quality control assessment using FastQC confirmed uniform base quality scores, balanced nucleotide composition, and absence of adapter contamination. Together, these metrics indicate high sequencing quality and support the reliability of downstream transcriptomic and pathway analyses.

**Figure 1:**
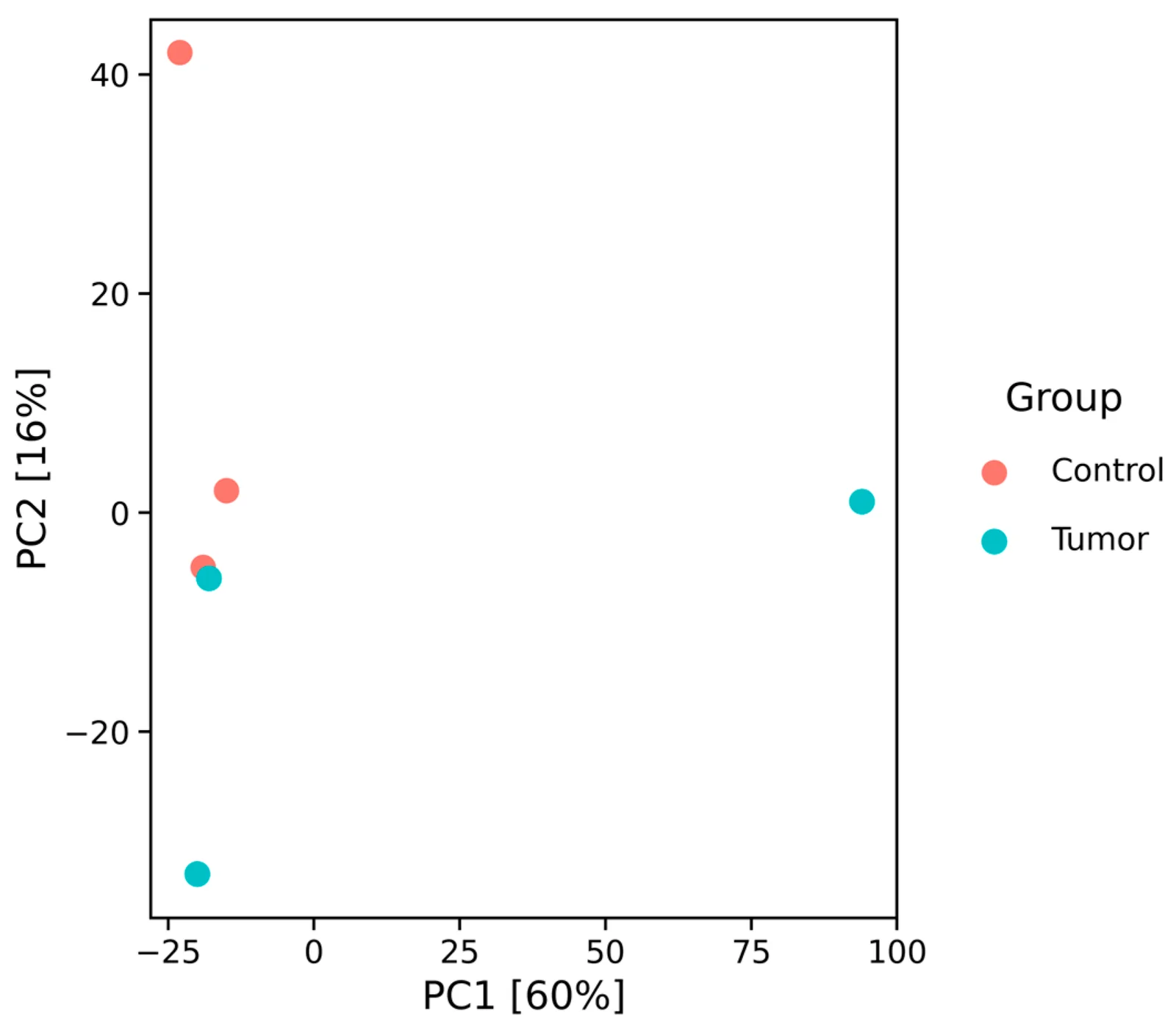
PCA of Leydig cell tumor and control samples based on the normalized expression matrix.

To investigate the molecular characteristics of canine Leydig cell tumors, we performed transcriptome analysis using RNA sequencing data derived from three Leydig cell tumors and three healthy Leydig cell samples. Principal component analysis (PCA) demonstrated a separation between tumor and control samples along the first principal component (PC1), which accounted for 60% of the variance (Figure 1). The second principal component (PC2) explained 16% of the variance and captured sample-level variability within each group.

**Figure 2:**
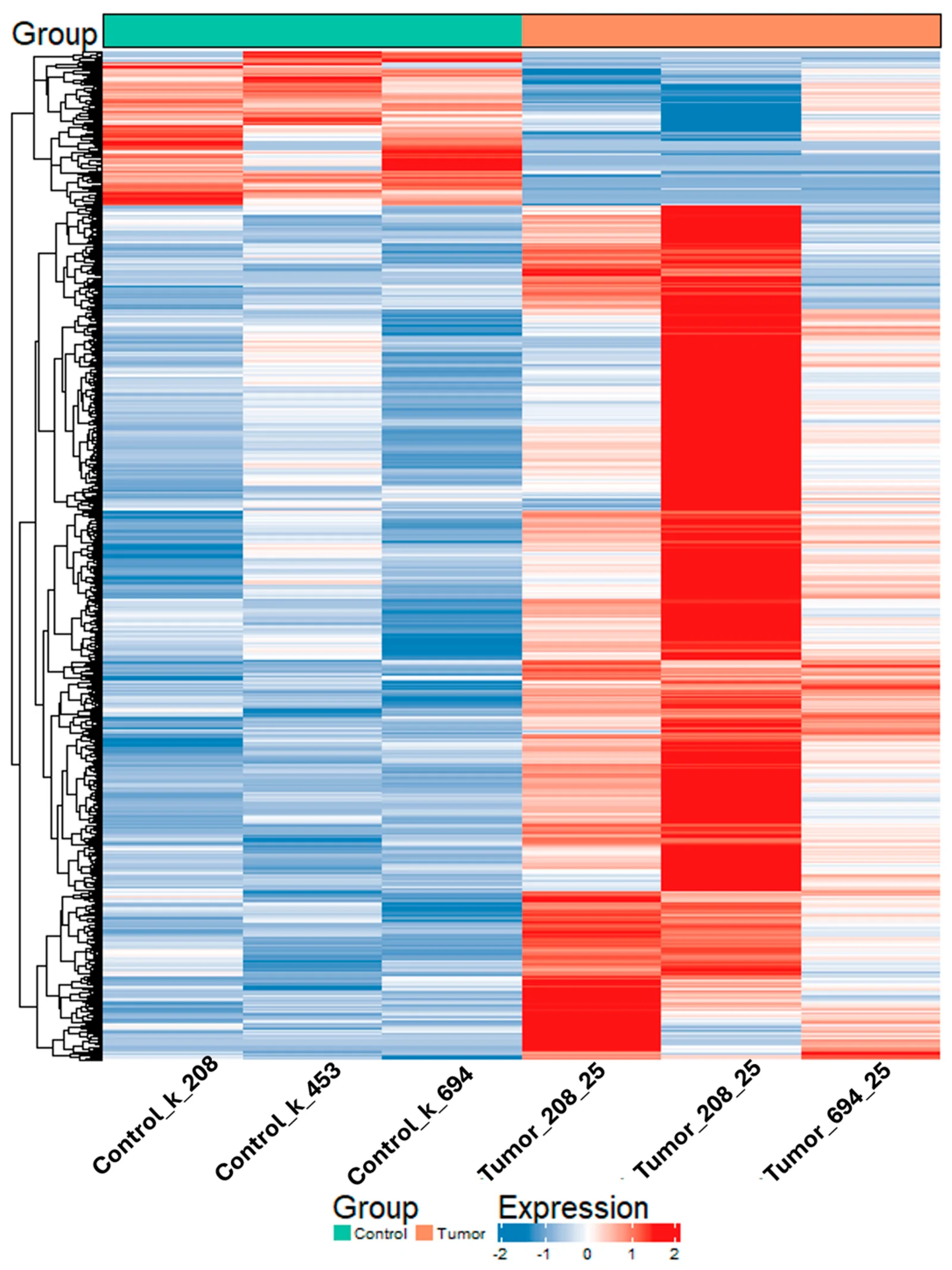
Heatmap of the top 1000 most variable genes (z-score scaled).

Hierarchical clustering and heatmap visualization of the top 1000 most variably expressed genes further highlighted a distinct transcriptional signature between Leydig cell tumor and control (Figure 2). Tumor samples exhibited consistent upregulation of specific gene clusters, suggesting coordinated transcriptional regulation in these Leydig cell populations.

**Figure 3:**
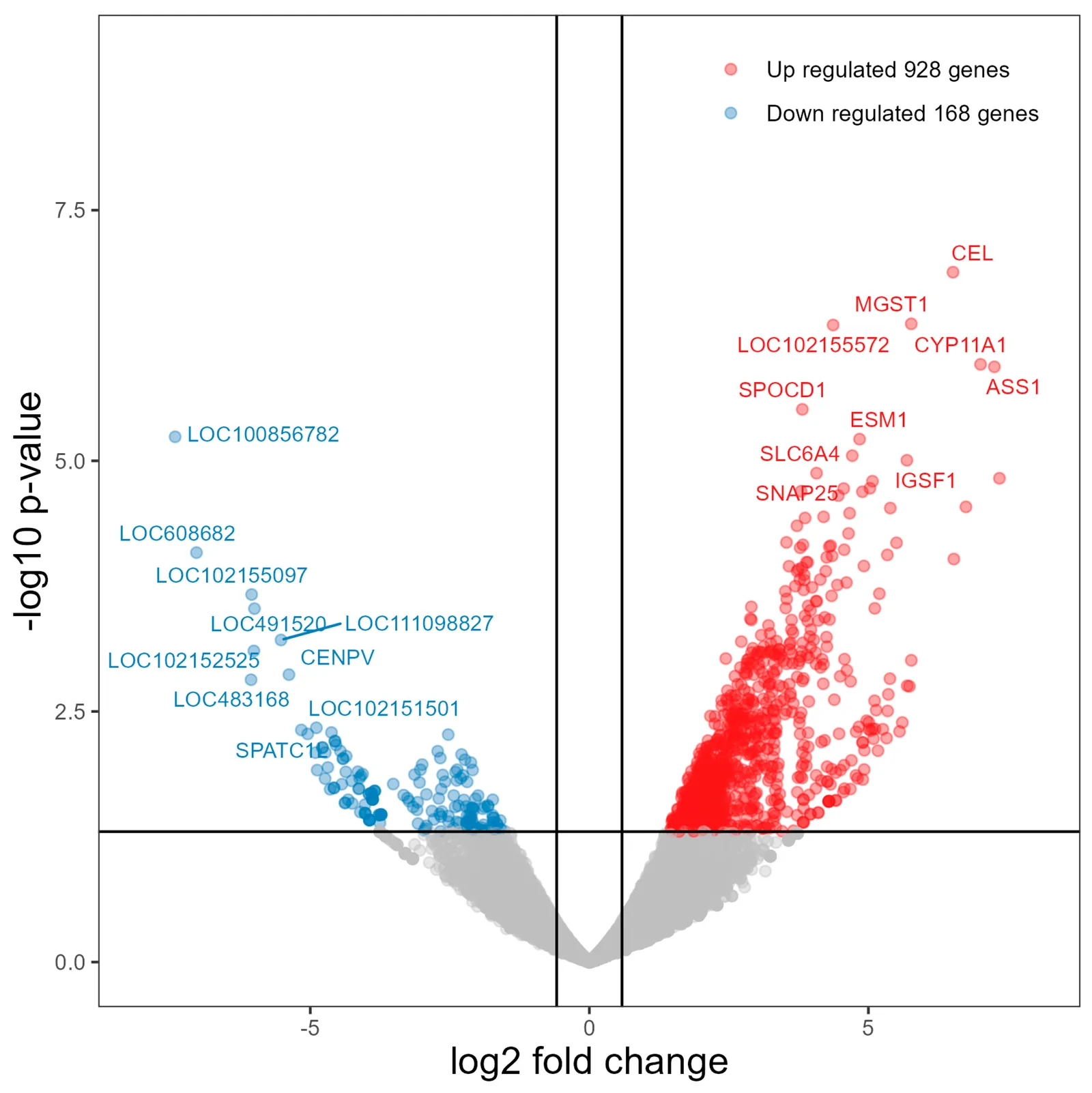
Volcano plot showing differentially expressed genes; red: upregulated (log2FC > 1.5), blue: downregulated (log2FC < −1.5), *p* < 0.05.

Differential expression analysis identified a total of 1500 transcripts with *p*-value < 0.05 and absolute log2 fold change ≥ 1.5. Of these, 928 genes were significantly upregulated, while 168 genes were significantly downregulated in tumor samples compared to controls (Figure 3).

### 3.2. Upregulated Genes in Canine Leydig Cell Tumor

Analysis of the most significantly upregulated genes in canine Leydig cell tumors revealed distinct transcriptional signatures associated with steroidogenesis, angiogenesis, neuroendocrine signaling, and metabolic adaptation. Among the most highly expressed transcripts were key regulators of steroid hormone biosynthesis, including *CYP11A1*, *STAR*, and *HSD3B1*, together with additional steroidogenic markers such as *CYP17A1*, *LHCGR*, and *INSL3*. These genes represent core components of Leydig cell function and constitute one of the most prominent expression patterns observed in tumor tissues.

A second major group comprised genes involved in vascular development and remodelling. Increased expression of *VEGFA*, *ESM1*, *FGG*, *KDR*, *ANGPT2*, and *CD93* indicated activation of angiogenesis-associated programs and vascular reorganization within the tumor microenvironment. Consistent with this observation, several extracellular matrix- and stromal-related genes, including *COL1A1*, *COL1A2*, *POSTN*, and *LOXL2*, were also upregulated.

Notably, canine Leydig cell tumors additionally displayed elevated expression of genes linked to neuroendocrine and neurotransmitter-related signaling pathways. These included *SLC6A4*, *GRIN2C*, *GRIA1*, and *GABRB3*, suggesting transcriptional alterations in signaling mechanisms not typically associated with normal testicular physiology. Furthermore, several genes involved in cholesterol metabolism and cellular redox homeostasis, such as *DHCR24*, *MSMO1*, *FDPS*, and *GPX3*, were significantly overexpressed. Collectively, these findings demonstrate that canine Leydig cell tumors are characterized by coordinated activation of steroidogenic, vascular, stromal, and metabolic transcriptional programs.

### 3.3. Downregulated Genes in Canine Leydig Cell Tumor

Among the significantly downregulated transcripts in canine Leydig cell tumors, several genes with known or potential relevance to testicular biology and tumor regulation were identified. These include arachidonate 12-lipoxygenase and arachidonate 5-lipoxygenase; *ALOX12* and *ALOX5*, ankyrin repeat and SOCS box containing 16 gene; *ASB16*, chromogranin A; *CHGA*, chemokine-like factor; *CKLF*, DMRT like family C2 gene; *DMRTC2*, DNA meiotic recombinase 1; *DMC1*, epithelial cell adhesion molecule; *EPCAM*, FAT atypical cadherin 1; *FAT1*, *MIR8905*, non-SMC condensin I complex subunit H; *NCAPH*, paternally expressed gene 10; *PEG10*, preferentially expressed antigen in melanoma; *PRAME*, PNMA family member 6F; *PNMA6F*, semaphorin-3C; *SEMA3C*, and spermatogenesis associated 46 gene; *SPATA46*. The observed altered expression of these genes may reflect changes in cell differentiation programs, altered cell signaling, or impaired immunoregulatory mechanisms in the tumor microenvironment.

### 3.4. Functional Enrichment Analysis of Differentially Expressed Genes

To elucidate the biological processes dysregulated in canine Leydig cell tumors, we conducted pathway enrichment analysis using KEGG and GO databases. Enriched terms were filtered based on a *p*-value < 0.05.

**Figure 4:**
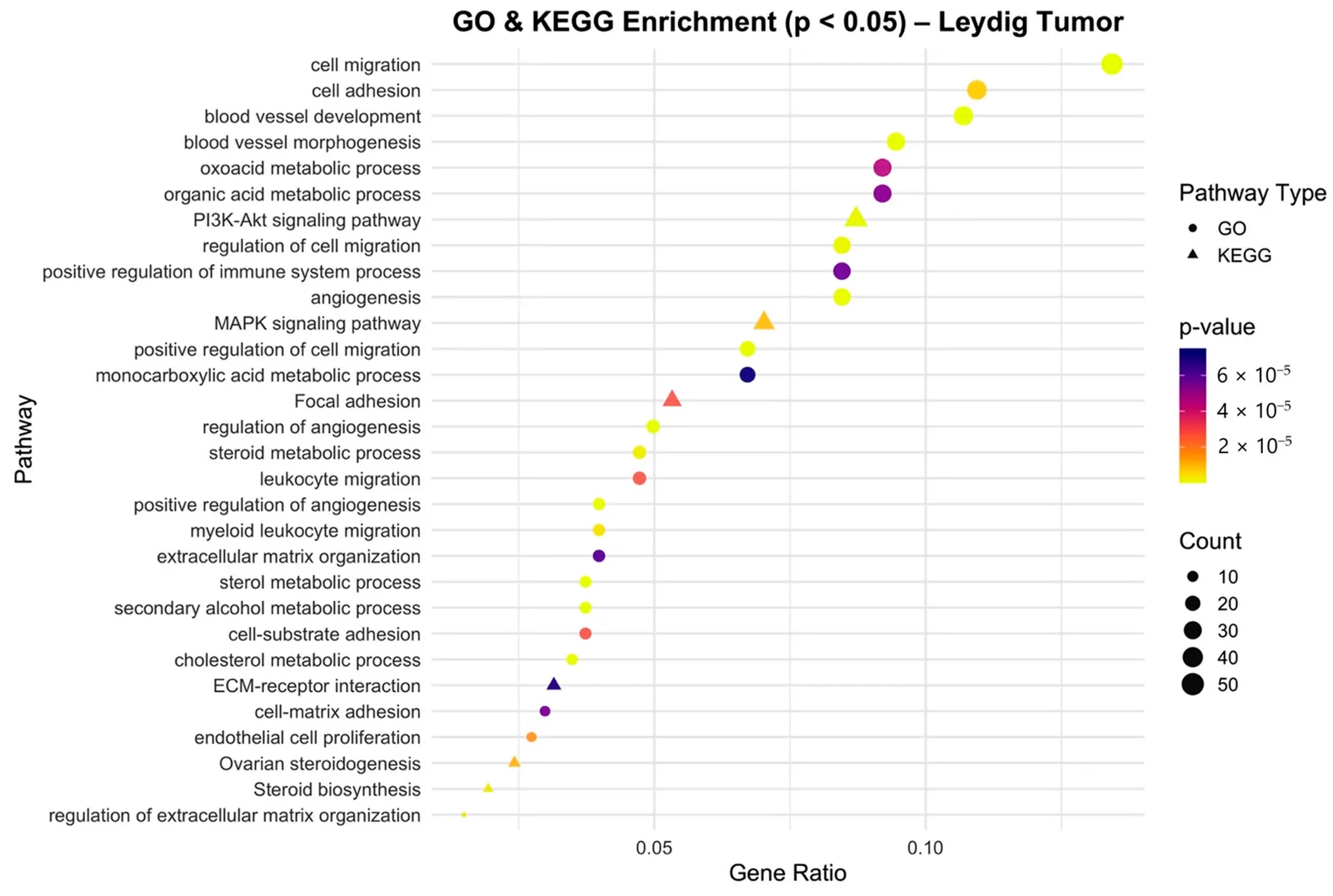
Dot plot representing enriched GO and KEGG pathways in Leydig cell tumor. Shape indicates pathway source (circle = GO, triangle = KEGG), color indicates *p*-value, and size reflects gene count.

Upregulated genes were predominantly associated with pathways related to steroid biosynthesis, PI3K-Akt signaling, extracellular matrix-receptor interaction, focal adhesion, and angiogenesis, reflecting the endocrine and invasive nature of Leydig tumors. GO enrichment analysis further highlighted cell migration, blood vessel morphogenesis, and immune-related processes, which may contribute to tumor progression (Figure 4).

Among the KEGG-enriched pathways, ovarian steroidogenesis, steroid biosynthesis, and the PI3K-Akt signaling pathway emerged as particularly relevant to the altered steroidogenic function and proliferative capacity of Leydig cell tumors.

**Figure 5:**
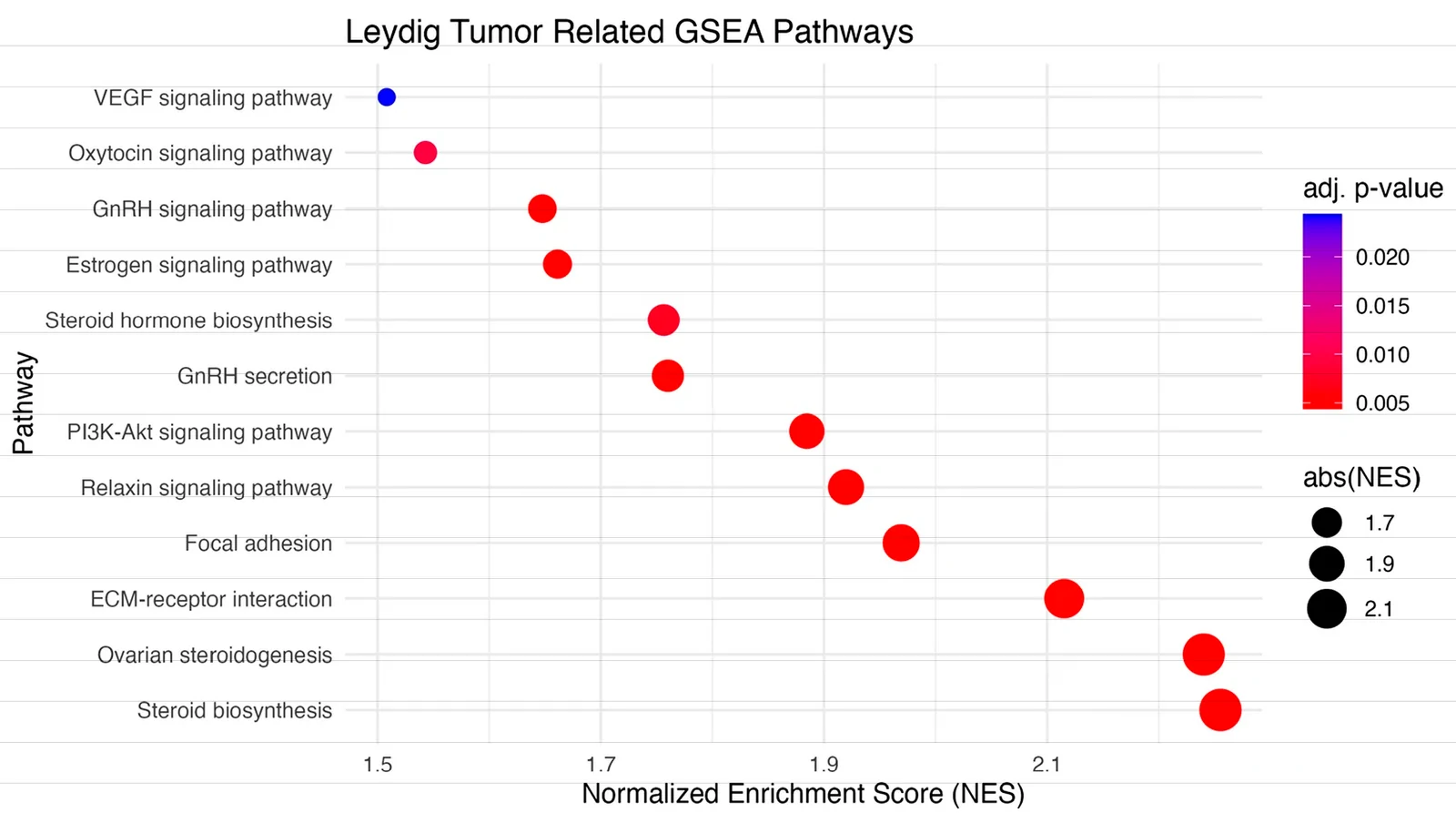
GSEA Dot Plot showing enriched pathways in canine Leydig cell tumor. Dot size represents the absolute normalized enrichment score (NES), and color denotes *p*-value significance. Key pathways involved include hormone biosynthesis, reproductive signaling, and extracellular matrix remodeling.

Gene Set Enrichment Analysis (GSEA) revealed several significantly enriched pathways in canine Leydig cell tumors compared to controls (Figure 5). Notably, pathways related to steroid hormone biosynthesis, ovarian steroidogenesis, and PI3K-Akt signaling were among the top-ranked. In addition, pathways including estrogen signaling, relaxin signaling, and GnRH-related cascades showed consistent enrichment, suggesting involvement of broader endocrine regulation. Interestingly, extracellular-receptor interaction and focal adhesion also emerged as significantly enriched, implying alterations in interstitial tissue structure, remodeling, cell communication, and immune response in the tumorigenesis course.

## 4. Discussion

Herein, data demonstrate that canine Leydig cell tumor pathogenesis is based on 1500 dysregulated genes being driven by a combination of a highly conserved oncogenic core shared with human Leydig cell tumors, such as tumorigenic signaling and progression, as well as alterations in steroidogenesis and local neuro-hormonal interactions. Concurrently, a severe suppression of developmental and immunoregulatory pathways uncovers how to resist systemic physiological barriers.

### 4.1. Disruption of Lipid Metabolism, Steroidogenesis, and Neuroendocrine Signaling

The primary physiological function of Leydig cells centers on the homeostasis of the biosynthesis of steroid hormones. Alterations in local stimuli (e.g., hormones, growth factors, and temperature) may create favorable conditions for the initiation and development of hyperplasia of Leydig cells [60]. The altered Leydig cell function is first manifested by fertility perturbations or sterility [42]. Only a deep understanding of the molecular mechanisms underlying these pathways is critical for these increasingly global reproductive health problems concerning also animals that may have an environmental background.

Our results demonstrate that HPG axis dynamics and downstream regulation are profoundly deregulated in canine Leydig cell tumors, supporting tumor progression via the upregulated transcripts *SLC6A4*, *GRIN2C*, and *GABRB3* [61,62,63]. Central neuroendocrine regulations and local cross-talk across neural, vascular, and endocrine systems are shifted within the testicular interstitial compartment, transmitting aberrant signals to the seminiferous tubules [64]. Nowadays, in the most studied human Leydig cell tumor, morphological transitions and lipid metabolism disturbances have been reported [65], suggesting a high tumor potential for lipid metabolism, growth, and metastasis [66,67,68,69].

The upregulated lipid-metabolism transcripts *GPX3*, *MSMO1*, and *DHCR24* reveal that the initial stages of sex hormone biosynthesis are disrupted at multiple regulatory checkpoints, e.g., fetal gonad formation, tumorigeneses in accessory glands [70,71]. Moreover, in line with Relovska et al. [72], *DHCR24* maintains sterol homeostasis tightly linked to sperm mitochondrial sheath formation, directly impacting fertility. This metabolic rewiring promotes dysregulated steroidogenesis, as evidenced by the robust upregulation of *CYP11A1*, *HSD3B1*, and *StAR* [73,74,75,76]. Hyperactivated StAR enhances the rate-limiting transport of cholesterol across the inner mitochondrial membrane, leading to fluctuating, atypical endocrine profiles [77,78,79,80,81,82]. These hormonal imbalances account for the localized testicular degeneration, prostatic hyperplasia, or azoospermia frequently observed in canine patients with Leydig cell tumor [83,84].

### 4.2. Angiogenesis, Tubulogenesis, and Microenvironmental Adaptations

A molecular understanding of tubulogenesis could lead to new ways of diagnosing and treating tumors. It is worth noting that testicular tumor cells develop mechanisms that increase genetic diversity [85]. Consequently, facilitate adaptations under a variety of conditions, including hypoxia, nutrient deprivation, exposure to DNA-damaging agents, and immune responses [86]. Substantial portions of the upregulated genes identified in this study are associated with tube morphogenesis and active angiogenesis, indicating a high capacity for tumor growth and environmental adaptation (Supplementary Figures S1 and S2). Upregulated *FGG* modulates endothelial cell function, angiogenesis, and chronic inflammation [87]. Concurrently, *ESM1* acts as a potent vascular oncogene, promoting cell proliferation, migration, and invasion via Vascular Endothelial Growth Factor (VEGF) and PI3K-Akt-mTOR signaling [88,89,90]. In studied tumors, upregulated *VEGFA* orchestrates neovascularization. Our findings in canine Leydig cell tumors align with Reddy et al. [91], correlating with clinically observed hypervascularity in the testicular veins of humans and rodents. Under pathological conditions, excessive VEGFA suppresses the antitumor activity of local immune cells, and the pattern of vascularity is a characteristic feature of Leydig cell tumors [92].

### 4.3. The Convergent Role of the PI3K-Akt Axis and Extracellular Matrix Remodeling

In canine Leydig cell tumors, mirroring human counterparts, the PI3K-Akt pathway is highly misregulated alongside relaxin and estrogen networks [83,93,94,95,96,97,98,99,100,101,102,103,104,105], (for the estrogen issue in detail, please see the next section). The PI3K-Akt cascade serves as a central oncogenic driver in endocrine cancers [96]. In these tumors, hyperactivation of the PI3K-Akt pathway closely interacts with local hormone signaling, modulating hormone production while simultaneously enhancing cell metabolism and cytoskeletal dynamics crucial for cancer cell survival. Crucially, the PI3K-Akt axis operates synergistically with altered extracellular matrix interactions. For instance, in male dogs, very low INSL3 concentrations (0.02–0.46 ng/mL), when compared to humans, were reported [5]. However, under pathological conditions as found here, perturbation of relaxin (family peptide to INSL3) signaling can be related to angiogenesis during remodeling of various tissues [103,104,105]. Therefore, it is likely that weak INSL3 regulation in canine testicular functions, like in immature or ageing mammals, allows for compensation by relaxin signaling [106]. Relaxin regulates collagen remodeling in endocrine tissues, which is related to estrogen levels [107,108]. This may further result in cancer-associated fibrosis (called paradoxical) [109,110]. It is possible that relaxin also regulates tumor Leydig cell invasion and migration during metastasis, which is primarily directed to different organs in humans and dogs [56,111,112,113]. This structural multidirectional remodeling, precisely accomplished by matrix metalloproteinases, removes the physical boundaries that control Leydig cell functional status, allowing tumor cells to release focal adhesions and initiate local tissue infiltration [114].

### 4.4. Distinctive Species Mechanisms: Estrogen Signaling

In a healthy male dog, serum estradiol concentrations range from approximately 5 to 25 pg/mL (low or even reference-range concentrations do not exclude biologically significant hyperestrogenism) [83]. Estrogen signaling is deeply implicated in the morpho-functional pathological alterations of tumor Leydig cells in dogs, which show in cytology vacuolization due to lipid accumulation [83], and in humans [95,96,97,101]. Secreted by affected Leydig cells, high estrogen levels feminize the male, suppress bone marrow, and may lead to benign prostate hyperplasia. Negative feedback on the HPG axis suppresses gonadotropin release in dogs, resulting in functional regression of the contralateral testis that may be predisposed to various clinical symptoms and pathological processes. Found here, for the first time, the involvement of estrogen signaling in canine tumor development points to a need for cautious interpretation in conjunction with further research and clinical findings. Overexpression of estrogen synthase (aromatase) or structural alterations in receptor expression disturb the local hormone milieu, leading to Leydig cell hyperplasia and cell engulfment by macrophages [66,76,101,102,103]. The presence or absence of estrogenic effect has a marked impact on the prognosis of Leydig cell tumors [83,100,102]. The identified alterations in estrogen signaling genes, correlating with aberrant clinical estrogen levels in dogs with Leydig cell tumors, warrant further research to develop genetic screening tools for veterinary practice. Such an approach would enable early diagnosis and risk prediction of Leydig cell tumor development later in the dog’s life.

### 4.5. Downregulation of Critical Developmental and Defensive Transcripts

The malignant phenotype of canine Leydig cell tumor is ultimately solidified by the silencing of a portion of genes involved in development, cell differentiation, adhesion, and immunoregulation. Transcripts dedicated to restricting aberrant vascularization and promoting normal differentiation, such as *DMRTC2*, *SEMA3C*, *DMC1*, *NCAPH*, and *PEG10* were profoundly downregulated, which indicates a total loss of local differentiation control [115,116,117,118]. Simultaneously, the silencing of immunoregulatory and cell-adhesion transcripts like *ALOX12*, *ALOX15*, *CKLF*, *EPCAM*, and *FAT1* highlights a successful evasion of host immune surveillance [119,120]. Obtained data on the portion of genes with decreased expression confirm that the processes they control are a conserved mammalian strategy enabling tumor progression [121]. This significantly advances our understanding of canine Leydig cell tumors, establishing the dog tissues as an invaluable comparative model for studying interstitial cell tumorigenesis in mammals [55]. The proximity of control sample k_453 and tumor sample 694_25 in the PCA may reflect biological heterogeneity among canine Leydig cell tumors. Differences in cellular composition, steroidogenic activity, and individual genetic background may contribute to partial transcriptomic overlap between specific tumor and control samples, despite the overall tumor-specific expression pattern observed across the dataset.

## 5. Conclusions

Collectively, this pioneering characterization of the altered transcriptome in canine Leydig cell tumors maps an interconnected molecular network of dysregulated genes. Utilizing tissues from mixed-breed dogs provides a spontaneous Leydig cell tumor model that circumvents breed-specific genetic biases. Importantly, this approach reflects the complex genetic background of the human population, enabling a cautious bidirectional translation of data with special account of species-specific differences, such as coexisting pathologies and distinct histological or immunological profiles. Despite these variations, existing data on Leydig cell tumors are limited to human research, which remains the closest comparative framework for canine research and clinical practice. Furthermore, our findings highlight that tumor expression mechanisms can vary among individual canine cases. This variability is driven by intra-tumor heterogeneity comprising distinct cellular subpopulations with unique genotypes, phenotypes, and functional capacities, with possible environmental influences (e.g., endocrine-disrupting chemicals).

Obtaining comprehensive genetic data advances the current understanding and clinical practice in canine andrology. Furthermore, the identification of some conserved transcriptomic dysregulations in canine Leydig cell tumors provides a basis for advancing both veterinary oncology and comparative biomedical research. Additionally, to translate these baseline genetic findings into clinical value, future studies must link transcriptomic data with high-throughput quantitative proteomics. Clinically, exploiting specific expression patterns within the distinct relaxin, estrogen, and neuroendocrine pathways (particularly *SLC6A4* and *GABRB3*) offers a promising path for developing sensitive, early-stage diagnostic biomarkers. Furthermore, the systematic suppression of the PI3K-Akt signaling axis and key angiogenic drivers, such as *VEGFA* and *ESM1*, should be evaluated as targeted pharmacological interventions to disrupt the proliferation and migration of altered Leydig cells.

## Data Availability

Dataset available on request from the authors.

## Supplementary Materials

The following supporting information can be downloaded at: https://www.mdpi.com/article/10.3390/ani16132005/s1.
