# Supplementary figures and images for: Transcriptomic Profiling of Canine Testicular Leydig Cell Tumors Uncovers Key Upregulated Gene Pathways
Source: Animals (Basel). 2026 Jul 1;16(13):2005. doi: 10.3390/ani16132005 (PMC13359613; doi:10.3390/ani16132005)

Supplement 1

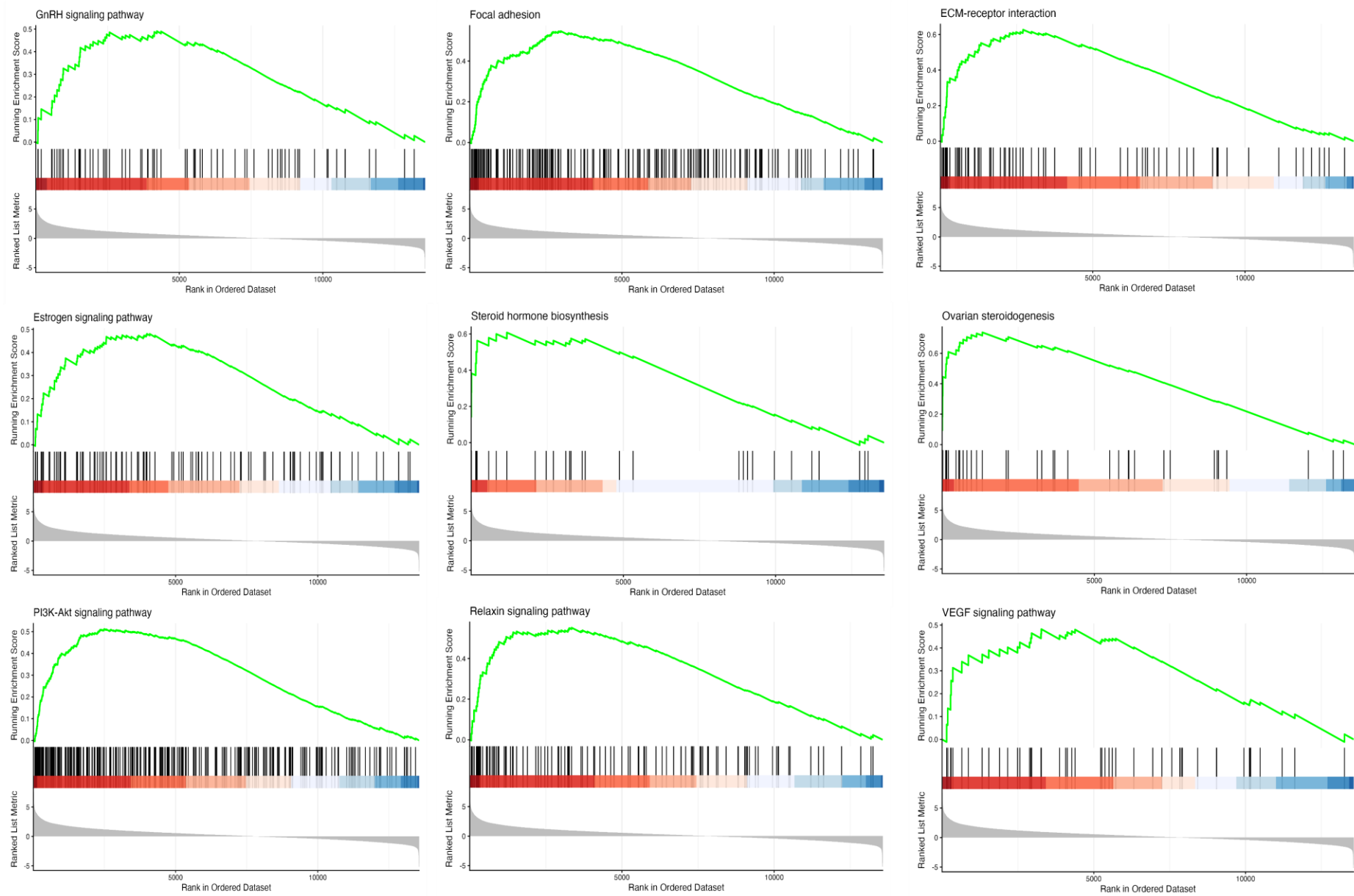

Supplement: Supplementary file 1 [file animals-16-02005-s001.zip › suppl_ Figure S1.pdf]

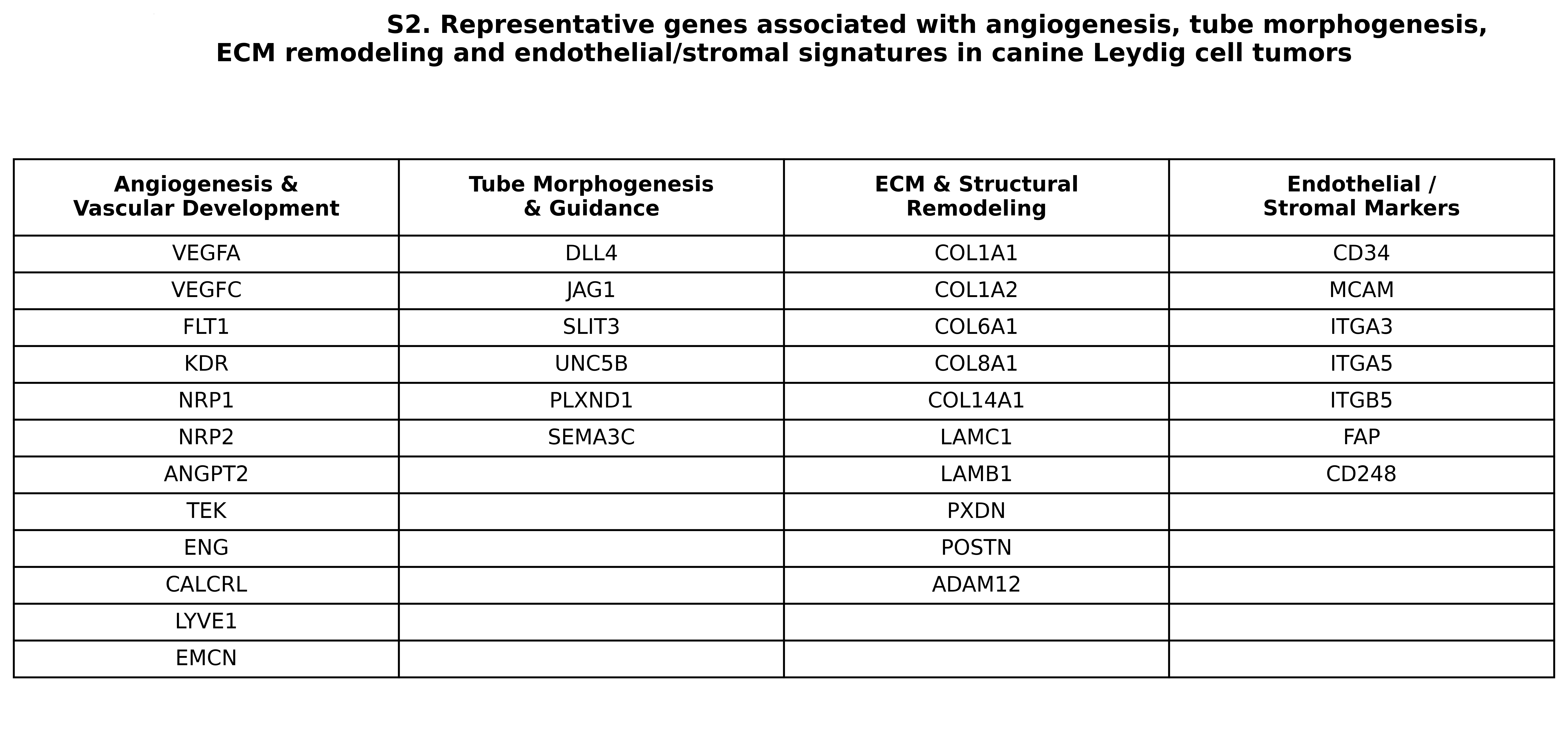

Supplement: Supplementary file 1 [file animals-16-02005-s001.zip › Suppl_Figure S2.png]
